# Phosphoproteomic Profiling of *In Vivo* Signaling in Liver by the Mammalian Target of Rapamycin Complex 1 (mTORC1)

**DOI:** 10.1371/journal.pone.0021729

**Published:** 2011-06-28

**Authors:** Gokhan Demirkan, Kebing Yu, Joan M. Boylan, Arthur R. Salomon, Philip A. Gruppuso

**Affiliations:** 1 Department of Pediatrics, Brown University and Rhode Island Hospital, Providence, Rhode Island, United States of America; 2 Department of Molecular Biology, Cell Biology, and Biochemistry, Brown University, Providence, Rhode Island, United States of America; 3 Department of Chemistry, Brown University, Providence, Rhode Island, United States of America; Texas A&M University, United States of America

## Abstract

**Background:**

Our understanding of signal transduction networks in the physiological context of an organism remains limited, partly due to the technical challenge of identifying serine/threonine phosphorylated peptides from complex tissue samples. In the present study, we focused on signaling through the mammalian target of rapamycin (mTOR) complex 1 (mTORC1), which is at the center of a nutrient- and growth factor-responsive cell signaling network. Though studied extensively, the mechanisms involved in many mTORC1 biological functions remain poorly understood.

**Methodology/Principal Findings:**

We developed a phosphoproteomic strategy to purify, enrich and identify phosphopeptides from rat liver homogenates. Using the anticancer drug rapamycin, the only known target of which is mTORC1, we characterized signaling in liver from rats in which the complex was maximally activated by refeeding following 48 hr of starvation. Using protein and peptide fractionation methods, TiO_2_ affinity purification of phosphopeptides and mass spectrometry, we reproducibly identified and quantified over four thousand phosphopeptides. Along with 5 known rapamycin-sensitive phosphorylation events, we identified 62 new rapamycin-responsive candidate phosphorylation sites. Among these were PRAS40, gephyrin, and AMP kinase 2. We observed similar proportions of increased and reduced phosphorylation in response to rapamycin. Gene ontology analysis revealed over-representation of mTOR pathway components among rapamycin-sensitive phosphopeptide candidates.

**Conclusions/Significance:**

In addition to identifying potential new mTORC1-mediated phosphorylation events, and providing information relevant to the biology of this signaling network, our experimental and analytical approaches indicate the feasibility of large-scale phosphoproteomic profiling of tissue samples to study physiological signaling events *in vivo*.

## Introduction

The mammalian target of rapamycin (mTOR) is a well conserved serine/threonine kinase that plays a key physiological role in the control of cell growth [1]. mTOR is a component of two distinct multiprotein complexes [2]–[4]. mTOR complex 1 (mTORC1) regulates temporal control of cell growth while mTOR complex 2 (mTORC2) regulates the organization of the actin cytoskeleton. mTORC1 signaling is sensitive to rapamycin, a macrolide antibiotic and anti-cancer drug, whereas mTORC2 mediates rapamycin-insensitive signaling [5]. mTORC1 signaling is stimulated by nutrients, growth factors, and high levels of cellular energy. In addition to the inhibitory effect of rapamycin, mTORC1 signaling is downregulated by environmental stressors, such as hypoxia and low cellular energy levels. Activation of mTORC1 leads to increased ribosome biogenesis, translation and nutrient transport, and to repression of autophagy and stress-induced transcription [1], [6].

The constituents of mTORC1 include mTOR, raptor, mLST8/G-protein β-subunit like protein (GβL), Proline-Rich Akt Substrate of 40 kDa (PRAS40), and Deptor [7]. This signaling complex exerts at least some of its biological effects by phosphorylating p70 ribosomal protein S6 kinase (S6K) at a single site, and eukaryotic initiation factor 4E (eIF4E) binding protein 1 (4E-BP1) at multiple sites. mTORC1 phosphorylation of critical residues involved in the activation of S6K1 (Thr389) and 4E-BP1 (Thr37 and Thr46) are generally sensitive to the inhibitory effects of rapamycin [8], [9]. Activated S6K1 functions *in vivo* to phosphorylate the 40S ribosomal protein S6, the biological significance of which is uncertain [10]. The phosphorylation of 4E-BP1 promotes its dissociation from eIF4E, thus activating 5′-cap-dependent mRNA translation, a process that accounts for the majority of total cellular translation [11]–[13]. Though much research has been devoted to understanding the mTORC1 pathway, the mechanisms underlying many of its biological functions remain poorly understood. It is likely that direct and indirect substrates of mTORC1 remain unidentified [1], [3], [12]–[16].

In the present study, we have utilized a well characterized model of mTORC1 activation *in vivo*, fasting followed by refeeding in the laboratory rat [17], [18]. During a period of food deprivation lasting 48 hours, liver mass and protein content decrease by approximately one quarter and one third, respectively. Within one hour of refeeding, marked mTORC1 activation occurs. Rapamycin injection prior to refeeding prevents this activation [18]. While several groups have undertaken phosphoproteomic profiling of liver tissue [19]–[23], none have used this approach to investigate rapamycin-sensitive phosphorylation events *in vivo*.

Recent advances in the technology of mass spectrometry (MS) permit the wide-scale analysis of cell signaling events. A number of studies have focused on tyrosine phosphorylation, an approach that has benefited from the ability to enrich for tyrosine phosphorylated peptides using peptide immunoprecipitation with anti-phosphotyrosine antibodies [24]–[26]. While equivalent antibodies for phosphoserine and phosphothreonine have been employed previously [27], these antibodies did not prove useful for profiling protein phosphorylation in a highly complex sample.

Our goal of profiling mTORC1 signaling required that we refine and adapt available methods, and establish the reproducibility of our analyses. We combined several standard methods for protein and peptide fractionation to reduce sample complexity, thereby improving the sensitivity of the MS analysis [22], [28]. We also employed a newer strategy, the enrichment of phosphopeptides by using metal oxide chromatography (MOC). In this approach, phosphorylated peptides are enriched based on their affinity for metal oxides such as zirconium dioxide [29], titanium dioxide [30] or aluminum hydroxide [31]. Our studies used the more recently developed methodology of titanium dioxide (TiO_2_) chromatography in the presence of lactic acid and 2,5-dihydroxy benzoic acid (DHB) [32]–[34].

Phosphoproteomic studies that have focused on signal transduction have largely been conducted using cell lines, and quantification of the greatest number of phosphorylation changes has been given primary importance over reproducibility of analysis. As an alternative to quantitative methods that employ isotope labeling, some investigators have employed “label-free” quantitation [35]. However, data on the validity of this method for tissue analysis are very limited. We therefore took the approach of analyzing multiple technical and biological replicate samples using a phosphoproteomic platform that employs automated desalting and reversed-phase separation of peptides in a highly consistent and reproducible manner [36]–[38]. All column elutions and loading steps are accurately replicated through computer control [39].

Using the experimental approach described above, we were able to identify more than three thousand unique phosphorylation sites with high reproducibility on an LTQ-Fourier-transform ion cyclotron resonance (FTICR) classic mass spectrometer. By investigating the aforementioned *in vivo* model of mTORC1 activation and inhibition, we were able to identify a number of well characterized mTORC1 targets as well as previously unknown candidate phosphorylation events. Global analysis of our data provided for a broad characterization of hepatic mTORC1 signaling *in vivo*.

## Methods

### Materials

Rapamycin was purchased from LC Laboratories (Woburn, MA). Antibodies directed towards ribosomal protein S6 and phosphorylated S6 (Ser^235/236^) were from Cell Signaling Technology, Inc. (Danvers, MA). Sephadex G-25 Coarse, Q Sepharose Fast Flow, SP Sepharose Fast Flow, and Resource 15S media were purchased from GE Healthcare Bio-Sciences Corp. (Piscataway, NJ). Sequencing grade trypsin was from Promega Corp. (Madison, WI). Oasis HLB extraction cartridges were from Waters Corp. (Milford, MA). The Resource S column was purchased from GE Healthcare, Inc. (Piscataway, NJ). Titansphere Phos-TiO kits were obtained from GL Sciences, Inc. (Torrance, CA).

### Animal Studies, and Preparation and Processing of Liver Homogenates

Adult male Sprague-Dawley rats (Charles River Laboratories, Wilmington, MA) were used for all studies. In order to maximally activate signaling through mTORC1 [17], rats were fasted for 48 hr, at the end of which time they were administered vehicle (dimethyl sulfoxide; DMSO) or rapamycin (50 µg/g body weight) by intraperitoneal injection. Fifteen minutes after injection, a 1 hr refeeding period was initiated by replacing the food in their cages. All rats were sacrificed by exsanguination under pentobarbital sodium anesthesia (50 µg/g by intraperitoneal injection). Livers were flash-frozen in liquid nitrogen and stored at −70°C until use. Three rats per condition were studied. All animal studies were carried out in strict accordance with the recommendations in the Guide for the Care and Use of Laboratory Animals of the National Institutes of Health. The protocol was approved by the Institutional Animal Care and Use Committee of Rhode Island Hospital (CMTT# 0066-10).

Rat liver homogenates were prepared in 10 ml/g wet weight of Buffer A (50 mM β-glycerophosphate, pH 7.4, 150 mM NaCl, 100 µM sodium orthovanadate, 10 mM NaF, 1 mM EGTA, and 5 mM EDTA) using a glass/Teflon homogenizer (7 strokes at 700 rpm). Homogenates were centrifuged at 1,000× *g* for 15 min. Supernatants were removed and centrifuged at 100,000× *g* for 1 hr. The resulting pellets were discarded. The supernatants retained and stored at −70°C.

Subsequent sample preparation was carried out at 4°C. Upon thawing, homogenate (9 ml) was mixed with 18 ml of Buffer B (150 mM NaCl, 100 µM sodium orthovanadate, 10 mM NaF, 1 mM EGTA, 5 mM EDTA). The diluted homogenate (25 ml) was applied to a 60 ml Sephadex G-25 Coarse column equilibrated in Buffer C (10 mM Tris, pH 8.6, 100 µM sodium orthovanadate, 10 mM NaF, 0.5 mM EGTA, 2 mM EDTA). The column was eluted with 60 ml Buffer C. Fractions containing protein were collected and stored at −70°C until further analysis.

In order to fractionate homogenates into multiple samples for further analysis, strong anion exchange (SAX) separation was carried out on the gel filtered sample (50 mg protein) using a 9.4 mm×25 mm column packed with Q Sepharose Fast Flow beads. Following adsorption of the sample, the column was washed at 0.8 ml/min with 5 ml of Buffer C. The flow-through was collected. The column was then eluted stepwise at 0.8 ml/min with 5 ml of Buffer C containing 150 mM, 300 mM or 1 M sodium chloride. The SAX flow-through was adjusted to pH 7.3 then applied to a strong cation exchange (SCX) SP Sepharose column (9.4 mm×25 mm). The column was washed at 0.8 ml/min with Buffer D (Buffer C adjusted to pH 7.3) and the flow-through (30 ml) was collected. The column was then eluted with 10 ml of Buffer D containing 1 M NaCl. This combination of SAX and SCX chromatography yielded a total of 5 fractions.

### Peptide Preparation and Fractionation, and Purification of Phosphopeptides

Protein concentrations in the five fractions were measured using Bradford reagent. Samples containing 50 mg protein were denatured by adding urea to a final concentration of 8 M and dithiothreitol to a final concentration of 1 mM, followed by incubation for 1 hr at 56°C. Samples were then alkylated with iodoacetemide (final concentration, 5 mM) followed by incubation for 1 hr at room temperature in the dark. These samples were diluted four-fold with 10 mM Tris, pH 8.0, and incubated overnight at 37°C with sequencing grade modified trypsin at a 1∶100 protease∶protein ratio. The resulting tryptic peptides were adjusted to pH 2.7 with trifluoroacetic acid, and then cleared by centrifugation at 1000× *g* for 5 min. The peptide mixtures were desalted using Oasis HLB extraction cartridges and dried in a SpeedVac Concentrator.

Dry peptides were reconstituted in Buffer E (5 mM ammonium formate, pH 2.65, 30% acetonitrile) and loaded onto a 1 ml Resource S (SCX) column equilibrated with Buffer E. The column was eluted with a linear gradient of 5 mM to 1.5 M ammonium formate (flow rate of 0.5 ml/min) using an ISCO ProTeam LC chromatography system. Including the flow-through, 32 fractions (1 ml each) were collected. The peptide content of each fraction was estimated by measuring the absorbance at 280 nm. As described below, two fraction pools containing the highest peptide amounts were recovered from each chromatogram. These pools were dried in a SpeedVac Concentrator and stored at −70°C.

For further processing, each peptide SCX pool was dissolved in 400 µl of 0.1% formic acid, 30% acetonitrile. These samples were each separated into four 100 µl samples for the performance of technical replicates. Phosphopeptides were enriched using Titansphere Phos-TiO reagents. Adsorption proceeded according to the manufacturer's protocol. Phosphopeptides were eluted from the Phos-TiO tips by first applying 25 µl of 1% ammonium hydroxide in water. The tips were then treated with 25 µl of 1% ammonium hydroxide in 40% acetonitrile and finally eluted with 50 µl of 5% pyrrolidine in water. All samples were dried in a SpeedVac Concentrator and stored at −70°C.

### Automated MS Analysis

Samples were analyzed by a fully automated phosphoproteomic technology platform that incorporates peptide desalting and separation via reverse phase chromatography followed by tandem mass spectrometry with static peak parking [40]. Briefly, dry phosphopeptide-enriched mixtures were dissolved in 0.1% acetic acid in water and adsorbed to a C18 analytical column (360 µm outer diameter×75 µm inner diameter; fused silica with 12 cm of 5 µm Monitor C18 particles and an integrated 4 µm ESI emitter tip fritted with 3-µm silica; Bangs Laboratories, Fishers, IN). Samples were eluted with a gradient of 0–70% 0.1 M acetic acid in acetonitrile that was developed over 30 min at a flow rate of 1.8 µl/min. Resulting eluates were applied directly to the mass spectrometer (Linear Trap Quadrupole-Fourier Transform [LTQ-FT]; Thermo Fisher Scientific, Waltham, MA). As described previously, static peak parking was performed via flow rate reduction from 200 nl/min to 20 nl/min once peptides began to elute as determined from a bovine serum albumin peptide scouting run [41]. The electrospray voltage of 2.0 kV was applied in a split flow configuration and spectra were collected in positive ion mode [39]. One full MS scan in the Fourier Transform (from m/z 400 to 1800) was followed by 9 data-dependent MS/MS spectra in the linear ion trap from the 9 most abundant ions. Selected ions were dynamically excluded for 30 s and screened for charge-states of +1, +2 and +3. All other settings for FTMS and ion trap mass spectrometry scans were the same as described previously [40].

### Data Analysis and Peptide Quantification

Custom software was used for the analysis of all data. Automation of database searches, statistical validation of peptide sequence, analysis for phosphorylation site position, and uploading to a relational database was handled by our High Throughput Autonomous Proteomic Pipeline (HTAPP) [38]. Quantitative proteomic data, including replicate analyses, was aggregated into heatmap representations using our PeptideDepot software [36].

Using our HTAPP software, MS/MS spectra were automatically searched against separate mouse, rat and hamster National Center for Biotechnology Information (NCBI) non-redundant protein databases (constructed on 7.30.2009) using the SEQUEST algorithm provided with Bioworks 3.2 (SEQUEST v.27 rev12). Peak lists were generated using extract_msn.exe version 4.0 using a mass range of 600–4500, precursor ion tolerance (for grouping) of 0.005 atomic mass unit, minimum ion count of 5, group scan of 0, minimum group count of 1. The NCBI rat, mouse and hamster databases contained 401,138 protein entries (50% forward, 50% reversed). SEQUEST was performed with the following parameters: trypsin enzyme specificity, 2 possible missed cleavages, 0.2 Da mass tolerance for precursor ions, and 0.5 Da mass tolerance for fragment ions. Search parameters specified a differential modification of phosphorylation (+79.9663 Da) on serine, threonine, and tyrosine residues and a static modification of carbamidomethylation (+57.0215 Da) on cysteine. To provide high confidence phosphopeptide sequence assignments, SEQUEST results were filtered by Xcorr (+1>1.5; +2>2.0; +3>2.5), precursor mass error (<20 ppm), and a logistic spectral score [37] that assessed MS/MS spectral quality (>0.965), minimum peak area threshold of 500. Non-redundant phosphopeptides and proteins with descriptors of “unnamed” or “unknown” were removed. False discovery rate was estimated with the decoy database approach after final assembly of nonredundant data into a comparison file [42]. To validate the position of the phosphorylation site, the Ascore algorithm [43] was applied to all data. The reported phosphorylation site position reflected the top Ascore prediction. Ascore probabilities are reported in the full data table (Table S1).

Quantitative analysis, collation of replicates, and visualization of proteomic data was accomplished using PeptideDepot software [36]. In order to compare peptide abundance among replicates, peak areas were calculated by inspection of selected ion chromatograms (SICs) using a software programmed in Microsoft Visual Basic 6.0 based on the Xcalibur Development Kit 2.0 SR2 (Thermo Fisher Scientific) [36]. This approach used the ICIS algorithm available in the Xcalibur XDK with the following parameters: multiple resolutions of 8, noise tolerance of 0.1, noise window of 40, scans in baseline of 5, and inclusion of refexc peaks parameter value, which is false. Retention time alignment was performed to correct chromatographic shifts between runs. In brief, commonly observed peptides in each pair of liquid chromatography/mass spectrometry (LC/MS) runs were divided into 25 groups, evenly sorted by their retention time at 1% false discovery rate. The most abundant peptide within each group was selected as a landmark to align two MS runs. Quantitative data were calculated automatically for every assigned peptide in all LC/MS runs based on exact precursor mass and retention time. For the case in which a peptide was not confidently identified by MS/MS spectrum in a given LC/MS experiment, its retention time was predicted using the retention time observed in other LC/MS runs where the peptide was identified confidently through an MS/MS spectrum after alignment correction.

A label-free comparison data file was generated for the evaluation of phosphopeptide abundance in DMSO and rapamycin treated animals (Table S1). Based on an approach in which samples from control and rapamycin-treated animals were processed and analyzed in parallel, all comparisons were paired. That is, label-free ratios corresponding to peptide abundance differences between paired control and rapamycin animals for 3 biological replicate sets were calculated. Blanks in the data columns indicated that a clearly defined SIC peak was not observed for that phosphopeptide in any of the technical replicate analyses for that particular sample. The coefficient of variation was calculated for each animal sample among the 4 technical replicate analyses and 3 biological replicate analyses (Table S1).

A threshold for rapamycin effect was set at 5-fold. This degree of change was based on an analysis of the control∶rapamycin ratios for all phosphopeptides (Table S2).

To classify proteins from the liver phosphoproteome and rapamycin-sensitive candidates, gene ontology and pathway terms were examined (www.geneontology.org) using the PeptideDepot software [36]. Classifications were based on the gene ontology slim terms and mapped to the Kyoto Encyclopedia of Genes and Genomes (KEGG) Pathway Database (http://www.genome.ad.jp/kegg/pathway.html). Phosphorylation site-specific kinase predictions were obtained using Kinexus' PhosphoNET kinase predictor software (http://www.phosphonet.ca).

## Results

### Method Development

**Figure 1:**
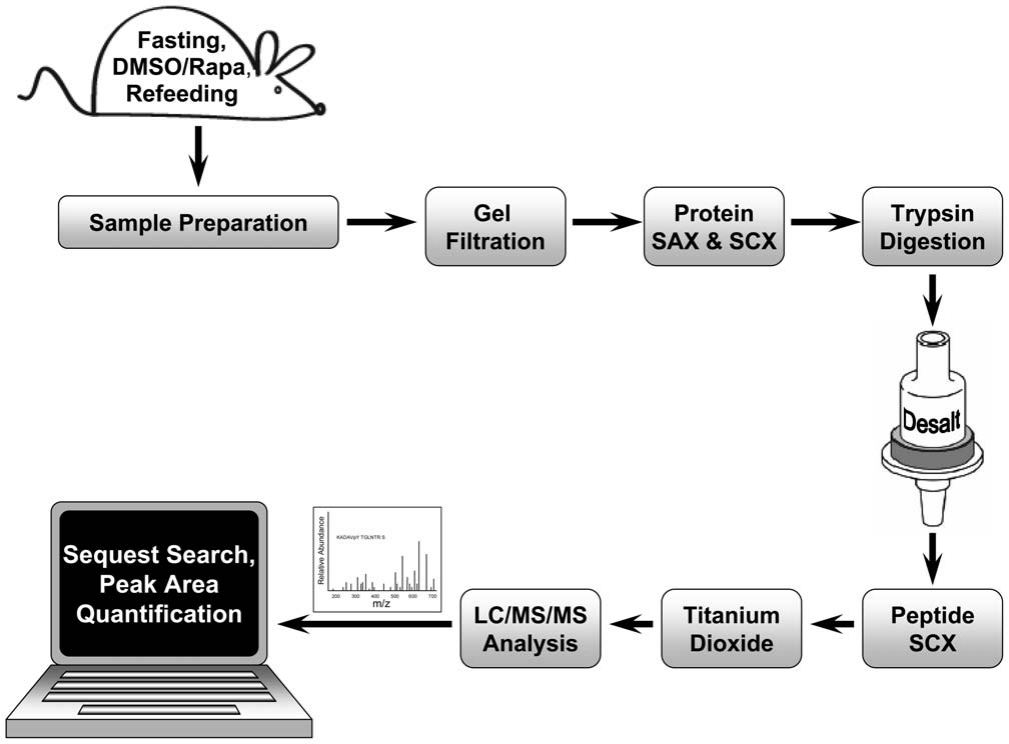
Experimental approach for the phosphoproteomic profiling of liver homogenates. Six rats were fasted for 48 hr, and then injected with DMSO or rapamycin. A 1 hr refeeding period was initiated 15 min after injection. As shown in this flow diagram, tissue homogenates were processed by sequential gel filtration, SAX and SCX chromatography. Samples were then reduced, alkylated, and digested into peptides. Tryptic peptides were desalted using Oasis HLB cartridges and further separated into two fraction pools by SCX chromatography. Phosphopeptides were then enriched by TiO_2_ chromatography. Purified phosphopeptides were analyzed by reversed-phase LC-MS/MS.

The profiling of mTORC1 signaling in liver required that we develop and validate methods for homogenate preparation, protein fractionation and peptide fractionation (Fig. 1). The first step in our strategy focused on removing phospholipids from rat liver homogenates. This was achieved by passing liver homogenates through a Sephadex G-25 gel filtration column. Gel electrophoresis and protein quantification showed greater than 90% protein recovery after this step.

In order to fractionate the sample to enhance MS phosphopeptide detection, ion exchange chromatography was performed following gel filtration. First, SAX chromatography with step elution generated a flow-through fraction and fractions containing proteins eluted with 0.15 M NaCl, 0.3 M NaCl and 1 M NaCl. The SAX flow-through was applied to a SCX column, resulting in 2 additional fractions, a SCX flow-through fraction and the proteins eluted at 1 M NaCl. SDS-PAGE of the resulting ion exchange fractions (not shown) demonstrated distinct patterns of protein expression. As intended, protein content of the five fractions was similar.

**Figure 2:**
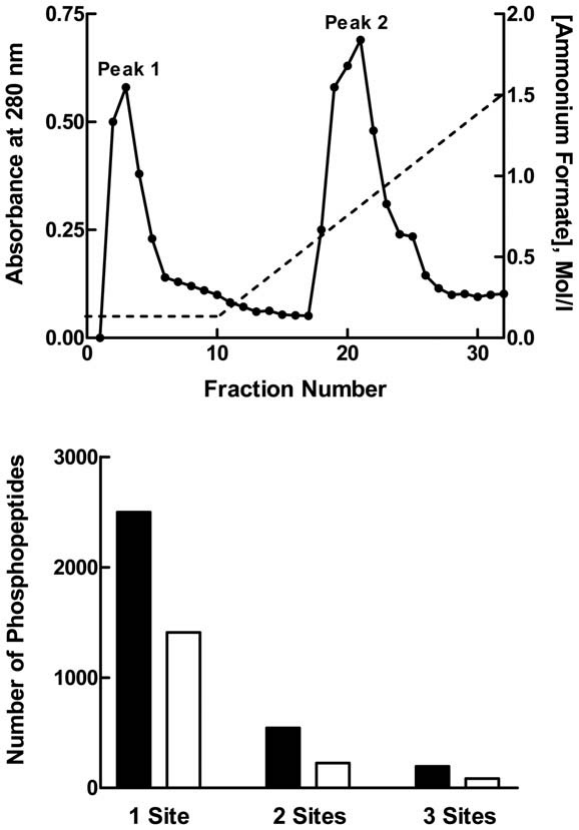
Phosphopeptide enrichment by SCX Chromatography. *Upper Panel*: Peptide SCX chromatography separation at pH 2.65 of a liver homogenate fraction. Digested peptides (10 mg) were applied to a Resource S SCX column and eluted using a 5 mM to 1.5 M ammonium formate gradient. The peptide content of each fraction was estimated by measuring the absorbance at 280 nm. Two fraction pools containing the highest peptide amounts (Peaks 1 and 2) were recovered from each column run. The figure shows a representative result using the 300 mM NaCl SAX fraction of the control 1 liver sample. *Lower Panel*: The number of phosphopeptides identified from peak 1 (filled bars) versus peak 2 (unfilled bars) is shown as a function of whether those phosphopeptides contained one, two or three phosphorylation sites.

The five ion exchange fractions were digested with trypsin. The resultant peptides were desalted by solid-phase extraction and then further separated by peptide SCX chromatography (Fig. 2, *Upper Panel*). Two fraction pools containing the highest peptide amounts were collected from each column run. Each peptide SCX pool was separated into 4 samples for the performance of technical replicates. Phosphopeptides were enriched from each of these replicate pools by TiO_2_ affinity purification.

The efficacy of the peptide SCX chromatography was examined by comparing the phosphopeptides present in peak 1 versus peak 2. Since each phosphate group subtracts a net charge of 1 from a peptide at pH 2.65, we expected phosphorylated peptides to elute before non-phosphorylated peptides [44]. MS analysis confirmed that fractions from SCX peak 1 were highly enriched with phosphopeptides and successfully separated from the more complex, positively-charged, multivalent peptides that eluted in the second SCX peak (Fig. 2, *Lower Panel*). Following TiO_2_ chromatography, but prior to filtering of MS results for non-phosphorylated peptides, more than 90% of the peptides recovered from SCX peak 1 were phosphorylated. In comparison, the yield of phosphopeptides from SCX peak 2 was approximately 60%. Most importantly, this chromatography step further reduced the complexity of the samples for MS analysis.

The use of TiO_2_ affinity phosphopeptide enrichment was based on previous studies demonstrating its high phosphopeptide selectivity [30], [33]. TiO_2_ enrichment using loose TiO_2_ beads in the presence of 2,5-dihydroxybenzoic acid [30] provided for a modest improvement in phosphopeptide enrichment relative to initial analyses using Fe^3+^ immobilized metal affinity chromatography (IMAC). The lactic acid-treated Titansphere Phos-TiO kit was tested based on a report that it yields superior results [45]. A pyrrolidine solution along with 1% ammonium hydroxide solution [46] was also employed to further improve the efficiency of phosphopeptide elution from TiO_2_ beads.

Phosphopeptide enriched samples were analyzed by LC/MS as described above. The resulting MS/MS spectra (can be accessed at http://cellpathway.com/SupplementalMaterial3.zip and http://cellpathway.com/SupplementalMaterial4.zip) were searched against the mouse, hamster and rat subsets of NCBI non-redundant databases using the SEQUEST algorithm. Sequence assignments were made using stringent filtering criteria (logistic spectral score >0.965 [36], [40]; precursor mass error <20 ppm; minimum SIC peak area threshold of 500 for label-free quantitation). After filtering, the false discovery rate, estimated using a decoy database [20], [37], was 1%.

The final data set derived from three biological control replicates contained 3,231 nonredundant phosphorylation sites representing 1,400 proteins and derived from 4,238 phosphopeptides. To assess the reproducibility of our phosphopeptide enrichment strategy in the context of *in vivo* studies, we compared LC/MS runs from these three control biological replicates as well as four technical replicates within each biological replicate. LC/MS retention time alignments of multiple datasets were carried out to correct for chromatographic shifts between experiments using the PeptideDepot software. Eighty-two percent of the phosphopeptides were identified in all three control biological replicates. Only 13% and 5% of the phosphopeptides were identified in two and one of the control biological replicates, respectively. Analysis of technical replicates demonstrated that, on average, 40% of the phosphopeptides were identified in all four technical replicates in a given biological sample, while 27%, 20% and 13% of the phosphopeptides were identified in three, two and one of the technical replicates, respectively. Thus, the performance of the four technical replicates contributed greatly to the reproducibility of the biological replicates.

To evaluate the sensitivity of our method for relatively low abundance phosphopeptides, we examined the relationship between peak area and the probability of a phosphopeptide being detected in one, two or all three of the biological replicates. Results (not shown) confirmed that more abundant phosphopeptides were indeed identified with greater reproducibility.

### The Rat Liver Phosphoproteome

To characterize the rat liver phosphoproteome from animals in which hepatic signal transduction was nutrient-activated, we compiled a list of all identified phosphorylation sites generated by analysis of the six combined control and rapamycin-treated animals. A total of 3,234 unique phosphorylation sites representing 1,401 proteins and 4,241 phosphopeptides were identified from these samples (Table S1). Serine phosphorylation predominated (2,762 sites representing 85% of all phosphorylation sites). Serine sites were followed in abundance by threonine phosphorylation (415 sites; 13%) and tyrosine phosphorylation (57 sites; 2%). Close to certainty (>99%) of phosphorylation site localization was achieved for 71% of the data set (3,011 of 4,241 phosphopeptides). All phosphopeptides with ambiguous site assignments (Ascore <19) [43] are presented in a separate table (Table S1).

### Rapamycin-regulated Phosphorylation Events

**Figure 3:**
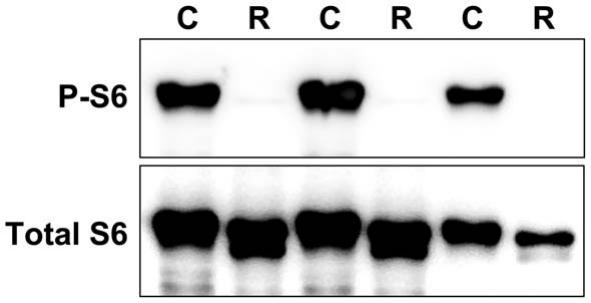
Demonstration of rapamycin *in vivo* effect. Extracts were prepared using liver samples from three control (C) and three rapamycin-treated (R) rats. The extracts (80 µg protein per lane) were analyzed by direct immunoblotting for phosphorylated ribosomal protein S6 (P-S6) and total S6.

Prior to performing the phosphoproteomic analysis, the efficacy of rapamycin administration was assessed by phospho-specific Western immunoblotting for ribosomal protein S6 (Ser235 and Ser236) [18]. Results (Fig. 3) showed a greater than 10-fold reduction in animals that were administered rapamycin prior to refeeding.

**Figure 4:**
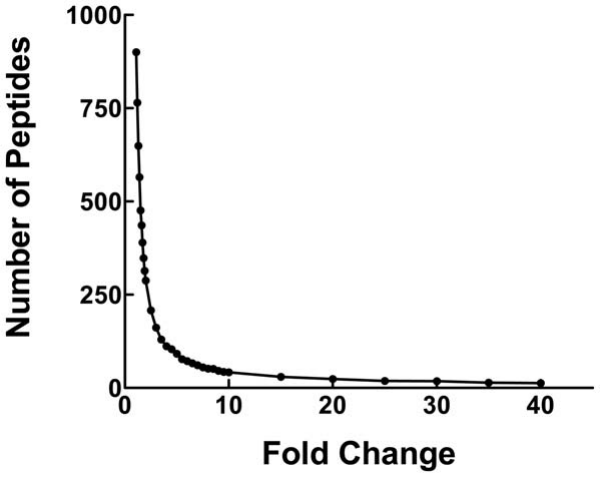
Distribution of rapamycin-induced fold-change in phosphopeptide abundance. For each peptide identified in all three control samples, fold-change in response to rapamycin was determined to be the minimum fold-change among the three paired analyses generated for each phosphopeptide. The graph shows the number of peptides in categories ranging from less than 1.1-fold different to 40-fold different or greater.

To identify rapamycin-modulated phosphorylation events involving soluble proteins from rat liver, we compared the results derived from animals that received DMSO vehicle versus rapamycin prior to refeeding. The magnitude of the phosphorylation change for each phosphopeptide in each paired analysis was determined using PeptideDepot software to generate mean peak area ratios. An analysis of the number of peptides showing graded degrees of change [47] showed an inflection point between 4-fold and 5-fold (Table S2 and Fig. 4). Similar to the approach taken by Chen et al. [48], we used this information to assign a conservative threshold of a 5-fold change as indicating an effect of rapamycin. That is, we applied a cutoff of >5 or <0.2 (ratio of control∶rapamycin) for sites whose abundance decreased or increased, respectively. Using these criteria, we identified 67 unique sites that were altered in response to rapamycin out of the total of 3,234 sites identified.

Of these 67 sites, 5 have been previously reported as downstream from mTORC1. These include Maf1 (Ser75) and raptor (Ser863), both of which were identified only recently [49], [50]. The other three rapamycin-sensitive sites were identified on the established mTORC1 downstream target, ribosomal protein S6. Consistent with immunoblotting results, S6 phosphorylation at Ser235 and Ser236 was reduced at least 10-fold in response to rapamycin in all three paired samples.

An unexpected observation was that the 62 phosphorylation sites not previously identified as rapamycin-sensitive were both up-regulated and down-regulated in association with rapamycin administration. Twenty-nine phosphorylation sites were reduced in abundance at least 5-fold following rapamycin administration (Table 1), while the other 33 rapamycin-sensitive phosphorylation sites were increased at least 5-fold in samples in response to rapamycin (Table 2). No tyrosine phosphorylated sites were identified as rapamycin-regulated.

**Table 1:** Phosphorylation sites down-regulated in response to rapamycin.

Protein name	Gene Symbol	Phosphosite	C/R Ratio 1	C/R Ratio 2	C/R Ratio 3
AKT1 substrate 1 (PRAS40) isoform CRA_d	AKT1S1	S203/S213	79	9	>100
AMP-activated protein kinase alpha-2	PRKAA2	S377	22	96	109
Autophagy-related protein 2 homolog A (rCG47388)	ATG2A	S1243	16	38	218
Bcl2-associated athanogene 3	BAG3	S174	12	>100	6
Carbomyl-phosphate synthase 2	CAD	S1859	9	>100	>100
Delta-4-3-ketosteroid 5-beta-reductase	AKR1D1	S235	>100	>100	>100
Eukaryotic translation initiation factor 3A (ZH12 protein)	EIF3A	S584	>100	>100	>100
Fam65a protein	FAM65A	S450	12	59	5
Family with sequence similarity 126, member B	FAM126B	T306	6	17	13
Growth factor receptor binding protein 7	GRB7	S364	21	>100	>100
Hepatoma-derived growth factor 3 (HDGF2)	HDGFRP2	S450	65	77	7
Liver regeneration-related protein LRRG07	AKR1C13	S129	7	14	8
MAF1 (Repressor of RNA polymerase III transcription)	MAF1	S75	11	29	37
mCG147067 protein [Mus musculus]	N/A	S76	>100	32	83
Metastasis suppressor 1, isoform CRA_a	MTSS1	S667	12	12	93
Peroxisome biogenesis factor 1	PEX1	S1181	7	8	30
Protein kinase C beta II	PRKCB	S660	80	21	14
Protocadherin alpha 4 homolog	PCDHA4	S145	>100	53	>100
PTPRF interacting protein (liprin) alpha 1	PPFIA1	S667	>100	14	12
Raptor	RPTR	S863	5	21	17
rCG21490, isoform CRA_a (LOC687565 protein)	LOC687565	T74	17	>100	>100
rCG50767 (Similar to La-related protein 4)	LOC683071	S379	26	>100	>100
Ribosomal protein S6	RPS6	S235/S236	>100	>100	>100
Ribosomal protein S6	RPS6	S235/S236/S240	>100	21	>100
Ribosomal protein S6	RPS6	S236/S240	51	87	>100
Similar to AI661453 protein (C6orf132)	RGD1561662	S680	5	9	49
Similar to hypothetical protein 4933430I17	LOC500475	S5S6	>100	>100	>100
Sugen Kinase 269 (hypothetical protein XP_236266)	RGD1312026	S281	>100	>100	>100
Ubiquitin specific peptidase 15, isoform CRA_a	USP15	S244	35	79	77
Ubiquitin specific peptidase 15, isoform CRA_a	USP15	S242	31	84	44
Ubiquitin specific protease 24	USP24	S1138	>100	27	21
WASH complex subunit FAM21 (NP61201)	FAM21C	S744	40	10	12

**Table 2:** Phosphorylation sites up-regulated in response to rapamycin.

Protein name	Gene Symbol	Phosphosite	C/R Ratio 1	C/R Ratio 2	C/R Ratio 3
Acyl-CoA thioesterase 1	ACOT1	S416	0.03	0.06	0.16
Ajuba protein	JUB	S147	<0.01	0.04	0.09
Arginase 1, liver	ARG1	T281	<0.01	0.01	0.01
Argininosuccinate synthetase	ASS1	T219	0.08	0.03	0.2
Carbohydrate responsive element binding protein	MLXIPL	S516	0.17	0.12	0.2
Cytosolic 3-hydroxy 3-methylglutaryl coenzyme A synthase	HMGCS1	S516	0.05	0.1	0.15
Cytosolic malate dehydrogenase	MDH1	(S188/S189)	<0.01	0.04	0.13
ERM-binding phosphoprotein (NHERF)	SLC9A3R1	S287T290/S291	0.07	<0.01	<0.01
Estrogen receptor-binding site associated antigen 9	EBAG9	S36	<0.01	0.07	0.02
Family with sequence similarity 83, member H	FAM83H	S871	0.01	0.15	0.1
GABA-B receptor-interacting scaffolding protein	AKAP9	S104	<0.01	0.08	0.11
GAPDH - Glyceraldehyde-3-phosphate dehydrogenase	GAPDH	T182	0.11	0.14	0.03
Gephyrin	GPHN	S200	<0.01	0.08	0.05
Inositol polyphosphate 5-phosphatase (Sec16 hom. A)	INNP5E	S711	<0.01	0.14	0.19
JTV1 protein (Aimp2)	AIMP2	T35	0.03	<0.01	<0.01
l-Afadin (AF6)	MLLT4	S1804	0.03	0.06	0.14
La ribonucleoprotein domain family, member 1	LARP1	S648	<0.01	0.19	0.2
La ribonucleoprotein domain family, member 1	LARP1	T644	0.01	0.19	0.2
Liver regeneration-related protein LRRG07	AKR1C13	S232	<0.01	0.13	0.08
Nuclear factor 1/A	NFIA	S280	0.06	0.02	0.03
Phosphoglucomutase 1	PGM1	T115	<0.01	<0.01	0.05
Phosphoglucomutase 1	PGM1	S117	<0.01	<0.01	0.03
Phosphoglycerate kinase 1	PGK1	S203	<0.01	0.1	0.01
Plakophilin 4	PKP4	S335	<0.01	0.14	0.14
rCG23015, isoform CRA_a [Rattus norvegicus] [107894 Da]	N/A	S356	0.01	0.06	0.03
rCG35745 (LOC679383 protein)	LOC679383	S469	0.17	<0.01	<0.01
Ribonuclease UK114 (perchrolic acid soluble protein)	HRSP12	S11	0.03	0.1	0.01
Ribonuclease UK114 (perchrolic acid soluble protein)	HRSP12	T10	<0.01	<0.01	<0.01
Ribosome binding protein 1	RRBP1	S1203	<0.01	0.18	<0.01
S6 protein kinase (p90-RSK1)	RPS6KA1	S363	0.04	0.15	0.05
Stromal interaction molecule 1	STIM1	S519	0.01	0.2	0.02

Several phosphorylation events were particularly noteworthy. Phosphorylation of AMP kinase 2 (AMPK2, Ser377), eukaryotic initiation factor 3a (eIF3a; Ser584), metastasis suppressor 1 (Ser667), protein kinase C beta (Ser660), and PRAS40 (Ser203, Ser213) were down-regulated in response to rapamycin. The protein kinase C beta site has been shown to regulate the cellular localization of this kinase [51]. Ser 363 on p90 S6 kinase 1, which was up-regulated, is one of the known, multiple phosphorylation sites required for activation of this kinase [52]. In addition, PRAS40 (Ser213) was previously shown to be phosphorylated by mTORC1 in vitro. However, phosphorylation of this site was shown not to be sensitive to rapamycin treatment in vivo by using mutated PRAS40 protein in HEK293 cells [53]. The remaining phosphorylation sites have not been characterized with regard to function, although gephyrin, PRAS40 and AMPK were shown previously to interact with mTORC1 [54]–[56].

### Gene Ontology and Pathway Analysis

We profiled the proteins identified in the phosphoproteomic analysis from control rat livers and compared this dataset to the phosphoproteins affected by rapamycin administration. To further characterize rapamycin action in liver, we used the Gene Ontology metadata in the Human Protein Reference Database (HPRD) [57]. For this analysis of rapamycin effect, we broadened the array of phosphoproteins by including those whose phosphorylation state changed by >5-fold in two of three biological replicates and at least 3-fold in the third replicate. This led to the categorization of 81 rapamycin-regulated candidate proteins.

**Figure 5:**
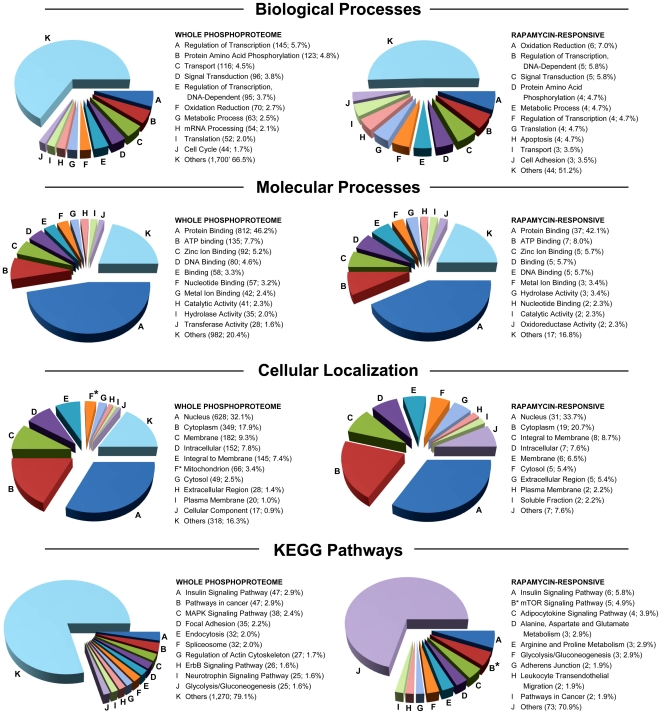
Gene ontology distribution of phosphoproteins in the total phosphoproteome and the rapamycin-responsive phosphoproteome. Distributions of the whole rat liver phosphoproteome are shown to the left for each analysis while the distributions of the phosphoproteins that were altered in response to rapamycin are shown on the right. The most abundant ten GO categories for each analysis are presented. Except where noted, an asterisk denotes a category that was significantly over-represented among rapamycin-sensitive candidate phosphoproteins as determined by chi-square analysis. Each category is followed by the number of genes identified in that category and the percentage of the total number of genes that were categorized for that analysis. For the cellular localization analysis, nine categories are presented for the rapamycin-responsive dataset since the same percentage values existed for the tenth and for all remaining categories. The asterisk denoting a whole phosphoproteome category indicates one that was significantly over-represented in that dataset. For the KEGG pathway categories, nine categories instead of ten are presented for the rapamycin-responsive dataset because the same percentage values were obtained for the tenth and multiple other categories.

Categorization of the rapamycin-responsive phosphoproteome for biological processes (Fig. 5, *top panel*) showed that 10.5% of the rapamycin-responsive candidate proteins were involved in the regulation of transcription, 7% in oxidation reduction, and 4.7% in translation. In the whole rat liver phosphoproteome, the same biological processes were similarly ascribed to 9.4%, 2.7%, and 2.0%, respectively. Categorization of phosphoproteins by cellular localization (Fig. 5. *third panel*) showed significant under-representation of the “mitochondrion” category among rapamycin-sensitive phosphoproteins. Categorization by molecular processes of all phosphoproteins detected (Fig. 5, *second panel*) showed dominance of “protein binding.” There were no significant differences when compared to the distribution of phosphoproteins that were affected by rapamycin.

KEGG Pathway analysis demonstrated that the phosphorylated proteins in the whole proteomic analysis were involved in 200 different pathways, including the insulin signaling pathway, pathways in cancer, and the mitogen-activated protein kinase (MAPK) signaling pathway. However, the set of rapamycin-sensitive proteins showed a significant over-representation of mTOR pathway constituents, which was the second most highly represented category after the insulin signaling pathway (Fig. 5, *bottom panel*). Based on the KEGG pathway analysis, the constituents of the mTOR pathway that were affected by rapamycin included raptor, ribosomal protein S6, AMP kinase 2, and the eukaryotic initiation factor eIF4b. Also included was p90S6 kinase 1, a component of the Erk signaling pathway that can be considered as involved in mTOR signaling based on cross-talk between pathways [51].

To further characterize candidate phosphorylation sites that were altered in response to rapamycin, we used Kinexus' PhosphoNET kinase predictor software (Table 3). In the human homologues of rat proteins, corresponding conserved phosphosites were searched. Sites on AMPK2 (Ser377), PRAS40 (Ser213), protein kinase C beta (Ser660) and raptor (Ser863), all of which were down-regulated in association with rapamycin administration, were strongly predicted to be targets of mTOR kinase. eIF3a (Ser584), hepatoma-derived growth factor 3 (Ser450), and PTPRF interacting protein (liprin) alpha 1 (Ser667) were predicted to be phosphorylated by p70S6 kinase. Two members of the phosphoinositide-3-kinase-related kinase family (of which mTOR is a member), ATM kinase and ATR kinase, were predicted to phosphorylate Bcl2-associated athanogene 3 (Ser174), Maf1 (Ser75) and peroxisome biogenesis factor 1 (Ser1181).

**Table 3:** Kinase substrate predictions.

Protein name	Gene Symbol	P-site	Predicted Kinase 1*	Predicted Kinase 2	Predicted Kinase 3	Predicted Kinase 4	Predicted Kinase 5
AKT1 substrate 1 (PRAS40) isoform CRA_d	AKT1S1	S203	CK2a1 (123)	CK2a2 (117)	p70S6K (106)	p70S6K (105)	SRPK1 (97)
AKT1 substrate 1 (PRAS40) isoform CRA_d	AKT1S1	S213	mTOR (247)	PCTAIRE3 (220)	PCTAIRE2 (214)	PCTAIRE1 (202)	ERK1 (200)
AMP-activated protein kinase alpha-2	PRKAA2	S377	mTOR (221)	NLK (170)	CDK1 (163)	CDK3 (161)	PCTAIRE3 (161)
Bcl2-associated athanogene 3	BAG3	S174	ATR (296)	ATM (245)	DNAPK (161)	SRPK1 (106)	SRPK2 (98)
Carbomyl-phosphate synthase 2	CAD	S1859	ROCK2 (195)	PIM1 (179)	ROCK1 (177)	MRCKb (171)	DMPK1 (169)
Eukaryotic translation initiation factor 3A (ZH12 protein)	EIF3A	S584	SGK2 (163)	PIM1 (163)	PKG1 (158)	AKT2 (156)	PIM3 (156)
Growth factor receptor binding protein 7	GRB7	S364	JNK1 (194)	JNK2 (194)	JNK3 (193)	mTOR ( 184)	MAPK14 (151)
Hepatoma-derived growth factor 3 (HDGF2)	HDGFRP2	S450	PIM1 (259)	PIM2 (256)	PIM3 (256)	p70S6K (249)	p70S6Kb (249)
MAF1 (Repressor of RNA polymerase III transcription)	MAF1	S75	ATR (309)	ATM (250)	DNAPK (135)	CK2a1 (61)	mTOR (55)
Peroxisome biogenesis factor 1	PEX1	S1181	ATR (343)	ATM (275)	PIM1 (172)	PIM3 (172)	PKG1 (169)
Protein kinase C beta II	PRKCB	S660	mTOR (136)	ILK (98)	MAPK12 (77)	ERK1 (69)	ERK2 (65)
PTPRF interacting protein (liprin) alpha 1	PPFIA1	S667	p70S6K (130)	p70S6Kb (130)	PIM2 (110)	PIM1 (109)	SGK (104)
Raptor	RPTR	S863	mTOR (293)	PCTAIRE3 (267)	PCTAIRE2 (263)	PCTAIRE1 (250)	CDK1 (232)
Ribosomal protein S6	RPS6	S235	PKCa (256)	PKCb (244)	ROCK2 (234)	MRCKb (231)	MRCKa (230)
Ribosomal protein S6	RPS6	S236	PIM1 (250)	p70S6K (249)	p70S6Kb (249)	PIM3 (247)	PIM2 (237)
Ribosomal protein S6	RPS6	S240	SRPK1 (162)	SRPK2 (156)	MSSK1 (151)	MRCKb (117)	MRCKa (116)
Sugen Kinase 269 (hypothetical protein XP_236266)	RGD1312026	S281	JNK2 (225)	JNK3 (219)	JNK1 (217)	PFTK1 (215)	MAPK14 (203)
Ubiquitin specific peptidase 15, isoform CRA_a	USP15	S242	mTOR (178)	ERK1 (155)	ERK2 (155)	PFTK1 (129)	ERK5 (126)
Ubiquitin specific protease 24	USP24	S1138	ATR (44)	ATM (42)	GSK3B (38)	PKACa (33)	SgK085 (33)

*Shown are the 5 human protein kinases that are most likely to phosphorylate each of the designated phosphosites. Kinase prediction scores are shown in parentheses. The higher the kinase prediction score, the better the prospect that a kinase will phosphorylate a given site.

## Discussion

In recent years, several groups have performed comprehensive tissue phosphoproteome analyses [19]–[23], [58]–[60]. The animal tissue most often used for phosphoproteome analysis has been liver [19]–[23]. The first such study, performed by Jin et al. [19], utilized iron IMAC for the enrichment of phosphorylated peptides and conventional linear ion-trap mass spectrometry (LTQ) for the phosphopeptide analysis. These investigators identified 26 nonredundant phosphorylation sites. A subsequent study [20] utilized high capacity iron IMAC and a higher mass accuracy MS Q-TOF instrument to identify 339 non-redundant phosphorylation sites from over 200 proteins. The analyses performed by Villen et al. in 2007 [21] was a breakthrough study. These investigators identified 5,635 nonredundant phosphorylation sites from 2,149 proteins, signifying the first in-depth global analysis of phosphopeptides from liver. Notably, these investigators used more selective iron IMAC beads and MS instruments of higher mass accuracy (the LTQ-FT and the LTQ-Orbitrap) as compared to previous studies. However, it is likely that the SCX pre-fractionation of tryptic peptides was a critical factor accounting for the increase in phosphopeptide identification.

Lack of reproducibility has been an issue with large scale MS-based phosphoproteomic profiling. Moser and White [20] tested the reproducibility of their methodology by performing three replicate analyses of the same rat liver homogenate. They observed that 56–63% (131 out of 207–234) of the peptides from each analysis were observed in all three analyses. Another issue is method of analysis. Alcolea et al. [61] found that analyzing the same phosphopeptide enriched murine NIH/3T3 fibroblast lysate by two different LC-MS/MS platforms based on Q-TOF and LTQ-Orbitrap mass spectrometers led to identification of partially overlapping, but also distinct, phosphoproteome profiles. The Q-TOF based platform resulted in 1,485 non-redundant phosphopeptide identifications, whereas the LTQ-Orbitrap based platform identified 4,308 non-redundant phosphopeptides. Only 1,077 of the total population of phosphopeptides were detected by both platforms. Analyzing duplicate samples by LC-MS/MS on the LTQ-Orbitrap platform showed that ∼70% of the identified phosphopeptides were identical. In a study comparing the peptides identified using two workflows, TiO_2_-SCX and SCX-TiO_2_, the overlap was 58 and 51% for the two methods, respectively [62]. A similar overlap of 60% was observed, when they performed replicate LC-MS/MS analyses of the same TiO_2_-SCX sample by an LTQ-Orbitrap.

These previous studies profiling the liver phosphoproteome have not been aimed at characterizing signaling events in a physiological context. Accomplishing this required improved sensitivity and accuracy, and a demonstration of reproducibility. We found that peptide abundance affects reproducibility, but that reproducibility could be enhanced by the performance of technical replicates. There are several indications that our methods were sufficient to detect mTORC1-mediated protein phosphorylation. These included the identification of known mTORC1 targets, the significant enrichment for mTOR signaling pathway constituents as indicated by pathway analysis, and the kinase prediction results.

In their seminal studies, Hunter and Sefton reported the relative abundances of pSer, pThr, and pTyr to be 90%, 10%, and 0.05%, respectively [63]. The order of magnitude higher tyrosine phosphorylation frequency in our results may be a function of the tendency for pTyr-containing peptides to yield better quality MS/MS spectra with collision-induced dissociation fragmentation. In contrast, phosphoserine and phosphothreonine containing peptides may produce low scores due to fragmentation patterns dominated by neutral loss of phosphate [21]. We are left to conclude that the actual proportion of protein phosphorylation accounted for by pTyr is between 0.05% and 2%, but likely toward the lower end of this range.

Two groups have recently reported large-scale, MS-based analysis of TORC1-dependent protein phosphorylation events [35], [48]. Chen et al. [48] used epidermal growth factor-induced HeLa cell cultures and employed the use of stable isotope labeling in cell culture (SILAC) to achieve quantitative analyses. They identified 250 rapamycin-sensitive phosphorylation sites from 161 cellular proteins. Their main finding was the identification of CDC25B (Ser375) as the key phosphorylation event in mediating rapamycin-induced oncogenic Akt activation [48]. Huber et al. [35] used Saccharomyces cerevisiae with various genetic backgrounds for label-free quantitative phosphoproteomic screens. This study reported 41 rapamycin-sensitive yeast proteins and revealed that rapamycin-regulated Sch9 (a homolog of mammalian kinases Akt and p70S6K1) is a central coordinator of protein synthesis.

To our knowledge, our work represents the first in-depth, global analysis of rapamycin-dependent phosphoproteomics performed on whole tissue samples. One noteworthy result is the identification of several rapamycin-sensitive candidates that are related to translation. eIF3a is the largest component of the eIF3 complex, which is required for several steps in the initiation of protein synthesis [64]. The eIF3 complex interacts with p70S6K under conditions of nutrient depletion or starvation [65]. We found that rapamycin administration was associated with a marked reduction in phosphorylation of a component of eIF3, eIF3a, at Ser584 in all three animal sets. Although the function of this site has not been defined, a search of the Minimotif Miner (http://mnm.engr.uconn.edu/) database reveals that the [KR]xRxx[ST] consensus motif is also present in ribosomal protein S6, a substrate for p70S6K. Our observation is consistent with the hypothesis that refeeding of rats after starvation causes the activation of mTORC1, leading to phosphorylation and release of p70S6K from the eIF3 complex.

We observed rapamycin-sensitive phosphorylation events involving several other proteins associated with translation. Ribonuclease UK114, also identified as “translational inhibitor protein p14.5”, has been shown in previous studies to be related to inhibition of cell proliferation [66], [67]. We found that rapamycin administration was associated with a >5-fold increase in phosphorylation of this protein at Thr10 and Ser11 in all three paired analyses. These sites have not been previously identified or characterized.

We also identified several known direct interactors of mTOR as rapamycin-sensitive phosphoproteins. Among these were the mTORC1 component raptor on Ser863 [49] and PRAS40, a novel mTOR binding partner (53). Rapamycin has been shown to decrease the association of PRAS40 with mTORC1 proteins (53), an event for which the mechanism has not been elucidated. The exact mechanism by which PRAS40 inhibits mTORC1 activity is not well understood. We found that rapamycin treatment was associated with reduced phosphorylation of rat PRAS40 at Ser203 and Ser213 in all three paired analyses. A recent phosphopeptide mapping study identified Ser183, Ser212 and Ser221 as mTOR-dependent phosphorylation sites in human PRAS40 [53]. Ser212 of human PRAS40, which is homologous to Ser213 in rat PRAS40, was not identified as sensitive to rapamycin treatment by these investigators.

AMPK is a critical sensor of metabolic stress that can turn off biosynthetic pathways when cellular ATP/AMP ratios decline [68]. The two AMP kinase isoforms, which generally function in the same manner (53), can inhibit mTORC1 by phosphorylating and activating TSC2. We detected a rapamycin-associated reduction in phosphorylation of AMPK2 at Ser377 in all three paired analyses. The Kinexus' kinase predictor software indicated that mTOR is a candidate kinase for this particular site. Our results may indicate a feedback mechanism between mTOR and AMPK through this rapamycin-sensitive phosphorylation event.

Gephyrin is a microtubule-associated protein involved in membrane protein-cytoskeleton interactions that is purported to directly interact with mTOR in a manner that is required for rapamycin-sensitive signaling [54]. The underlying mechanism has not been identified. We found that rapamycin administration was associated with a >12-fold increase in phosphorylation of gephyrin at Ser200, a site that has not been assigned a biological function.

Chen and coworkers detected more transcription-related proteins (9.3%) than translation-related proteins (5.4%) among their rapamycin-sensitive proteins [48], just as we did. However, the only rapamycin-sensitive phosphorylation events common to our dataset and theirs were the well-characterized ribosomal protein S6 sites. An RNA binding protein, the La ribonucleoprotein (LARP), was identified as a rapamycin-sensitive candidate in both studies. Phosphorylation of human LARP at Ser849, which is homologous to Ser648 of rat LARP, was reduced upon rapamycin exposure in the study by Chen et al. [48], while we observed an increase in the phosphorylation of rat LARP at the Thr644 and Ser648 sites in association with rapamycin administration. A comparison of our data to those of Huber and coworkers [35] shows that in both cases Maf1 and S6 were the only common rapamycin-sensitive proteins previously reported in the literature.

While our approach allowed us to identify what may be novel, physiologically relevant rapamycin-sensitive sites, our study has some important limitations. One is incomplete coverage of any given protein due to a suboptimal density of tryptic sites. For example, lysine and arginine content within eIF4G1 is very high, so digestion with trypsin results in very short peptide fragments. This likely accounted for our inability to identify peptides containing the rapamycin-sensitive Ser1108 phosphorylation site in eIF4G1 [69]. Other established rapamycin-sensitive phosphorylation sites include p70S6K (Thr389) and 4E-BP1 (Thr37) and (Thr46) [70]. We do not have a definitive explanation for the absence of p70S6K Thr389 in our analyses, though low abundance of this phosphoprotein is likely. While we detected the Thr37 and Thr46 phosphorylation sites in 4E-BP1, their phosphorylation was not affected by rapamycin in either our study or another published study [48]. This may be consistent with recent biochemical studies indicating a complex mechanism behind the effect of rapamycin on 4E-BP1 phosphorylation [48], [71], [72].

There are other limitations inherent in our approach. We began with a preparation of proteins that were soluble under aqueous, non-detergent-containing conditions. Since the presence of detergents in samples complicates MS analysis [73], [74], extension of our methods to lipid-soluble proteins may be challenging. Very low abundance proteins or proteins with a low stoichiometry of phosphorylation would not have been detected in our MS analysis due to sensitivity limits of the LTQ-FTICR classic mass spectrometer and the suppression of ionization efficiency with the detection of phosphorylated peptides in the positive ion mode [75]. These issues notwithstanding, we made another observation of potential physiological significance. That was the high frequency with which previously unidentified phosphorylation sites were up-regulated in association with rapamycin administration. It is, of course, the case that rapamycin-sensitive phosphoproteins may not be direct targets of mTOR kinase. The up-regulation of phosphorylation in response to rapamycin can be accounted for by a downstream kinase that is activated upon its own dephosphorylation. An example of such a mechanism is eukaryotic elongation factor 2 kinase (eEF2K), which is activated in response to its dephosphorylation [76]. Alternatively, such changes may reflect modulation of phosphatase activity.

Identification of potential rapamycin-sensitive phosphorylation sites is only the first step in characterizing the signaling events we have identified. The assignment of functional significance to newly identified sites will require a traditional biochemical approach. Nonetheless, through the application of a phosphoproteomics approach to an *in vivo* model of mTORC1 signaling we have identified 67 rapamycin-sensitive candidate phosphorylation events. Identification of known rapamycin-sensitive phosphorylation sites supported the reliability of our analysis. We identified a high number of novel rapamycin-sensitive candidate phosphorylation sites on proteins that are related to transcription, translation or cell growth, or are known to interact with mTOR kinase. While contributing to the understanding of mTOR action, our results indicate the potential utility of phosphoproteomic profiling of *in vivo* tissues in identifying new targets for drug therapies.

## Supporting Information

Table S1 **Table of peptide sequences, phosphorylation site assignment statistics (standard deviation and coefficient of variation), and quantitative analysis for all data described in this manuscript.**
(XLS)

Table S2 **Calculation of the inflection point for the distribution of rapamycin-induced fold-change in peptide abundance.**
(XLS)

## References

[1] Wullschleger S, Loewith R, Hall MN (2006). TOR signaling in growth and metabolism.. Cell.

[2] Alessi DR, Pearce LR, Garcia-Martinez JM (2009). New insights into mTOR signaling: mTORC2 and beyond.. Sci Signal.

[3] Dunlop EA, Tee AR (2009). Mammalian target of rapamycin complex 1: signalling inputs, substrates and feedback mechanisms.. Cell Signal.

[4] Guertin DA, Sabatini DM (2009). The pharmacology of mTOR inhibition.. Sci Signal.

[5] Abraham RT (2002). Identification of TOR signaling complexes: more TORC for the cell growth engine.. Cell.

[6] Hay N, Sonenberg N (2004). Upstream and downstream of mTOR.. Genes Dev.

[7] Foster KG, Fingar DC (2010). Mammalian target of rapamycin (mTOR): conducting the cellular signaling symphony.. J Biol Chem.

[8] Hidalgo M, Rowinsky EK (2000). The rapamycin-sensitive signal transduction pathway as a target for cancer therapy.. Oncogene.

[9] Loewith R, Jacinto E, Wullschleger S, Lorberg A, Crespo JL (2002). Two TOR complexes, only one of which is rapamycin sensitive, have distinct roles in cell growth control.. Mol Cell.

[10] Wool IG (1979). The structure and function of eukaryotic ribosomes.. Annu Rev Biochem.

[11] Gingras AC, Raught B, Sonenberg N (2004). mTOR signaling to translation.. Curr Top Microbiol Immunol.

[12] Ma XM, Blenis J (2009). Molecular mechanisms of mTOR-mediated translational control.. Nat Rev Mol Cell Biol.

[13] Nojima H, Tokunaga C, Eguchi S, Oshiro N, Hidayat S (2003). The mammalian target of rapamycin (mTOR) partner, raptor, binds the mTOR substrates p70 S6 kinase and 4E-BP1 through their TOR signaling (TOS) motif.. J Biol Chem.

[14] Choo AY, Blenis J (2009). Not all substrates are treated equally: implications for mTOR, rapamycin-resistance and cancer therapy.. Cell Cycle.

[15] Efeyan A, Sabatini DM (2010). mTOR and cancer: many loops in one pathway.. Curr Opin Cell Biol.

[16] Polak P, Hall MN (2009). mTOR and the control of whole body metabolism.. Curr Opin Cell Biol.

[17] Anand P, Gruppuso PA (2005). The regulation of hepatic protein synthesis during fasting in the rat.. J Biol Chem.

[18] Anand P, Gruppuso PA (2006). Rapamycin inhibits liver growth during refeeding in rats via control of ribosomal protein translation but not cap-dependent translation initiation.. J Nutr.

[19] Jin WH, Dai J, Zhou H, Xia QC, Zou HF (2004). Phosphoproteome analysis of mouse liver using immobilized metal affinity purification and linear ion trap mass spectrometry.. Rapid Commun Mass Spectrom.

[20] Moser K, White FM (2006). Phosphoproteomic analysis of rat liver by high capacity IMAC and LC-MS/MS.. J Proteome Res.

[21] Villen J, Beausoleil SA, Gerber SA, Gygi SP (2007). Large-scale phosphorylation analysis of mouse liver.. Proc Natl Acad Sci U S A.

[22] Han G, Ye M, Zhou H, Jiang X, Feng S (2008). Large-scale phosphoproteome analysis of human liver tissue by enrichment and fractionation of phosphopeptides with strong anion exchange chromatography.. Proteomics.

[23] Han G, Ye M, Liu H, Song C, Sun D (2010). Phosphoproteome analysis of human liver tissue by long-gradient nanoflow LC coupled with multiple stage MS analysis.. Electrophoresis.

[24] Salomon AR, Ficarro SB, Brill LM, Brinker A, Phung QT (2003). Profiling of tyrosine phosphorylation pathways in human cells using mass spectrometry.. Proc Natl Acad Sci U S A.

[25] Zhang Y, Wolf-Yadlin A, Ross PL, Pappin DJ, Rush J (2005). Time-resolved mass spectrometry of tyrosine phosphorylation sites in the epidermal growth factor receptor signaling network reveals dynamic modules.. Mol Cell Proteomics.

[26] Cao L, Yu K, Salomon AR (2006). Phosphoproteomic analysis of lymphocyte signaling.. Adv Exp Med Biol.

[27] Gronborg M, Kristiansen TZ, Stensballe A, Andersen JS, Ohara O (2002). A mass spectrometry-based proteomic approach for identification of serine/threonine-phosphorylated proteins by enrichment with phospho-specific antibodies: identification of a novel protein, Frigg, as a protein kinase A substrate.. Mol Cell Proteomics.

[28] Villen J, Gygi SP (2008). The SCX/IMAC enrichment approach for global phosphorylation analysis by mass spectrometry.. Nat Protoc.

[29] Kweon HK, Hakansson K (2006). Selective zirconium dioxide-based enrichment of phosphorylated peptides for mass spectrometric analysis.. Anal Chem.

[30] Larsen MR, Thingholm TE, Jensen ON, Roepstorff P, Jorgensen TJ (2005). Highly selective enrichment of phosphorylated peptides from peptide mixtures using titanium dioxide microcolumns.. Mol Cell Proteomics.

[31] Wolschin F, Wienkoop S, Weckwerth W (2005). Enrichment of phosphorylated proteins and peptides from complex mixtures using metal oxide/hydroxide affinity chromatography (MOAC).. Proteomics.

[32] Hubbard MJ, Cohen P (1993). On target with a new mechanism for the regulation of protein phosphorylation.. Trends Biochem Sci.

[33] Olsen JV, Blagoev B, Gnad F, Macek B, Kumar C (2006). Global, in vivo, and site-specific phosphorylation dynamics in signaling networks.. Cell.

[34] Thingholm TE, Jorgensen TJ, Jensen ON, Larsen MR (2006). Highly selective enrichment of phosphorylated peptides using titanium dioxide.. Nat Protoc.

[35] Huber A, Bodenmiller B, Uotila A, Stahl M, Wanka S (2009). Characterization of the rapamycin-sensitive phosphoproteome reveals that Sch9 is a central coordinator of protein synthesis.. Genes Dev.

[36] Yu K, Salomon AR (2009). PeptideDepot: flexible relational database for visual analysis of quantitative proteomic data and integration of existing protein information.. Proteomics.

[37] Yu K, Sabelli A, DeKeukelaere L, Park R, Sindi S (2009). Integrated platform for manual and high-throughput statistical validation of tandem mass spectra.. Proteomics.

[38] Yu K, Salomon AR (2010). HTAPP: high-throughput autonomous proteomic pipeline.. Proteomics.

[39] Cao L, Yu K, Banh C, Nguyen V, Ritz A (2007). Quantitative time-resolved phosphoproteomic analysis of mast cell signaling.. J Immunol.

[40] Nguyen V, Cao L, Lin JT, Hung N, Ritz A (2009). A new approach for quantitative phosphoproteomic dissection of signaling pathways applied to T cell receptor activation.. Mol Cell Proteomics.

[41] Ficarro SB, Salomon AR, Brill LM, Mason DE, Stettler-Gill M (2005). Automated immobilized metal affinity chromatography/nano-liquid chromatography/electrospray ionization mass spectrometry platform for profiling protein phosphorylation sites.. Rapid Commun Mass Spectrom.

[42] Elias JE, Gygi SP (2010). Target-decoy search strategy for mass spectrometry-based proteomics.. Methods Mol Biol.

[43] Beausoleil SA, Villen J, Gerber SA, Rush J, Gygi SP (2006). A probability-based approach for high-throughput protein phosphorylation analysis and site localization.. Nat Biotechnol.

[44] Beausoleil SA, Jedrychowski M, Schwartz D, Elias JE, Villen J (2004). Large-scale characterization of HeLa cell nuclear phosphoproteins.. Proc Natl Acad Sci U S A.

[45] Sugiyama N, Masuda T, Shinoda K, Nakamura A, Tomita M (2007). Phosphopeptide enrichment by aliphatic hydroxy acid-modified metal oxide chromatography for nano-LC-MS/MS in proteomics applications.. Mol Cell Proteomics.

[46] Kyono Y, Sugiyama N, Imami K, Tomita M, Ishihama Y (2008). Successive and selective release of phosphorylated peptides captured by hydroxy acid-modified metal oxide chromatography.. J Proteome Res.

[47] Bronshtein IN, Semendyayev KA, Musoil G, Muehlig H (2004). Handbook of Mathematics.

[48] Chen RQ, Yang QK, Lu BW, Yi W, Cantin G (2009). CDC25B mediates rapamycin-induced oncogenic responses in cancer cells.. Cancer Res.

[49] Foster KG, Acosta-Jaquez HA, Romeo Y, Ekim B, Soliman GA (2010). Regulation of mTOR complex 1 (mTORC1) by raptor Ser863 and multisite phosphorylation.. J Biol Chem.

[50] Shor B, Wu J, Shakey Q, Toral-Barza L, Shi C (2010). Requirement of the mTOR kinase for the regulation of Maf1 phosphorylation and control of RNA polymerase III-dependent transcription in cancer cells.. J Biol Chem.

[51] Keranen LM, Dutil EM, Newton AC (1995). Protein kinase C is regulated in vivo by three functionally distinct phosphorylations.. Curr Biol.

[52] Dalby KN, Morrice N, Caudwell FB, Avruch J, Cohen P (1998). Identification of regulatory phosphorylation sites in mitogen-activated protein kinase (MAPK)-activated protein kinase-1a/p90rsk that are inducible by MAPK.. J Biol Chem.

[53] Wang L, Harris TE, Lawrence JC (2008). Regulation of proline-rich Akt substrate of 40 kDa (PRAS40) function by mammalian target of rapamycin complex 1 (mTORC1)-mediated phosphorylation.. J Biol Chem.

[54] Sabatini DM, Barrow RK, Blackshaw S, Burnett PE, Lai MM (1999). Interaction of RAFT1 with gephyrin required for rapamycin-sensitive signaling.. Science.

[55] Cheng SW, Fryer LG, Carling D, Shepherd PR (2004). Thr2446 is a novel mammalian target of rapamycin (mTOR) phosphorylation site regulated by nutrient status.. J Biol Chem.

[56] Vander HE, Lee SI, Bandhakavi S, Griffin TJ, Kim DH (2007). Insulin signalling to mTOR mediated by the Akt/PKB substrate PRAS40.. Nat Cell Biol.

[57] Prasad TS, Kandasamy K, Pandey A (2009). Human Protein Reference Database and Human Proteinpedia as discovery tools for systems biology.. Methods Mol Biol.

[58] Xia Q, Cheng D, Duong DM, Gearing M, Lah JJ (2008). Phosphoproteomic analysis of human brain by calcium phosphate precipitation and mass spectrometry.. J Proteome Res.

[59] Zanivan S, Gnad F, Wickstrom SA, Geiger T, Macek B (2008). Solid tumor proteome and phosphoproteome analysis by high resolution mass spectrometry.. J Proteome Res.

[60] Liao L, McClatchy DB, Park SK, Xu T, Lu B (2008). Quantitative analysis of brain nuclear phosphoproteins identifies developmentally regulated phosphorylation events.. J Proteome Res.

[61] Alcolea MP, Kleiner O, Cutillas PR (2009). Increased confidence in large-scale phosphoproteomics data by complementary mass spectrometric techniques and matching of phosphopeptide data sets.. J Proteome Res.

[62] Wu J, Warren P, Shakey Q, Sousa E, Hill A (2010). Integrating titania enrichment, iTRAQ labeling, and Orbitrap CID-HCD for global identification and quantitative analysis of phosphopeptides.. Proteomics.

[63] Hunter T, Sefton BM (1980). Transforming gene product of Rous sarcoma virus phosphorylates tyrosine.. Proc Natl Acad Sci U S A.

[64] Mayeur GL, Fraser CS, Peiretti F, Block KL, Hershey JW (2003). Characterization of eIF3k: a newly discovered subunit of mammalian translation initiation factor elF3.. Eur J Biochem.

[65] Holz MK, Ballif BA, Gygi SP, Blenis J (2005). mTOR and S6K1 mediate assembly of the translation preinitiation complex through dynamic protein interchange and ordered phosphorylation events.. Cell.

[66] Morishita R, Kawagoshi A, Sawasaki T, Madin K, Ogasawara T (1999). Ribonuclease activity of rat liver perchloric acid-soluble protein, a potent inhibitor of protein synthesis.. J Biol Chem.

[67] Oka T, Tsuji H, Noda C, Sakai K, Hong YM (1995). Isolation and characterization of a novel perchloric acid-soluble protein inhibiting cell-free protein synthesis.. J Biol Chem.

[68] Kimura N, Tokunaga C, Dalal S, Richardson C, Yoshino K (2003). A possible linkage between AMP-activated protein kinase (AMPK) and mammalian target of rapamycin (mTOR) signalling pathway.. Genes Cells.

[69] Gruppuso PA, Tsai SW, Boylan JM, Sanders JA (2008). Hepatic translation control in the late-gestation fetal rat.. Am J Physiol Regul Integr Comp Physiol.

[70] Hall MN (2008). mTOR-what does it do?. Transplant Proc.

[71] Choo AY, Yoon SO, Kim SG, Roux PP, Blenis J (2008). Rapamycin differentially inhibits S6Ks and 4E-BP1 to mediate cell-type-specific repression of mRNA translation.. Proc Natl Acad Sci U S A.

[72] Dowling RJ, Topisirovic I, Alain T, Bidinosti M, Fonseca BD (2010). mTORC1-mediated cell proliferation, but not cell growth, controlled by the 4E-BPs.. Science.

[73] Thingholm TE, Jensen ON, Larsen MR (2009). Analytical strategies for phosphoproteomics.. Proteomics.

[74] Wu J, Warren P, Shakey Q, Sousa E, Hill A (2010). Integrating titania enrichment, iTRAQ labeling, and Orbitrap CID-HCD for global identification and quantitative analysis of phosphopeptides.. Proteomics.

[75] Lemeer S, Heck AJ (2009). The phosphoproteomics data explosion.. Curr Opin Chem Biol.

[76] Knebel A, Morrice N, Cohen P (2001). A novel method to identify protein kinase substrates: eEF2 kinase is phosphorylated and inhibited by SAPK4/p38delta.. EMBO J.

